# A Case of Late-Onset Acute Pulmonary Sarcoidosis Exacerbation After 26 Years of Clinical Stability

**DOI:** 10.7759/cureus.85111

**Published:** 2025-05-30

**Authors:** Yosuke Muarakami, Kazunori Tobino

**Affiliations:** 1 Respiratory Medicine, Iizuka Hospital, Iizuka, JPN; 2 Respiratory Medicine, Iizuka Hospital, Fukuoka, JPN

**Keywords:** acute pulmonary exacerbation, cryobiopsy, late-onset relapse, sarcoidosis, systemic corticosteroid therapy

## Abstract

Pulmonary sarcoidosis is a systemic granulomatous disease with a variable clinical course. While many cases resolve spontaneously, a subset of patients may develop chronic disease or experience acute pulmonary exacerbations. Such exacerbations typically occur within the first decade after diagnosis, and late-onset exacerbations after a prolonged quiescent phase are exceedingly rare.

We present the case of a 59-year-old Japanese man who developed acute pulmonary exacerbation of sarcoidosis (APES) 26 years after being diagnosed with stage I disease. He had remained clinically stable without systemic treatment and had only been followed for ocular involvement. He presented with progressive exertional dyspnea over four months, followed by fever and resting dyspnea. High-resolution computed tomography revealed bilateral ground-glass opacities, mediastinal lymphadenopathy, and traction bronchiectasis. Laboratory tests showed elevated lactate dehydrogenase (LDH), Krebs von den Lungen-6 (KL-6), and soluble interleukin-2 receptor (sIL-2R) levels. Bronchoalveolar lavage demonstrated lymphocytosis, and transbronchial lung cryobiopsy confirmed non-caseating granulomas. Infectious and autoimmune etiologies were excluded.

The patient was diagnosed with APES and treated with systemic corticosteroids, including an initial pulse therapy followed by tapering oral prednisolone. His oxygenation improved significantly, and supplemental oxygen was no longer required at rest by hospital day 24, although desaturation persisted with exertion. Follow-up imaging showed partial radiologic improvement, along with newly developed pneumomediastinum.

This case underscores the potential for sarcoidosis to reactivate even after decades of clinical remission. Long-term follow-up is essential, particularly in patients with fibrotic changes or extrapulmonary involvement. Early recognition and prompt management of late-onset APES may help prevent irreversible pulmonary damage.

## Introduction

Sarcoidosis is a multisystem inflammatory disorder characterized by the formation of non-caseating granulomas (small, organized collections of inflammatory cells that do not show tissue death at their center), with pulmonary involvement being the most common manifestation. Although the disease frequently follows a benign and self-limiting course, a subset of patients may experience chronic progression or acute exacerbations [1,2].

Acute pulmonary exacerbation of sarcoidosis (APES), a sudden worsening of lung-related sarcoidosis symptoms, is defined by progressive respiratory symptoms lasting at least one month and a decline of 10% or more in forced vital capacity (FVC, the total amount of air that can be forcibly exhaled after a maximal inhalation) and/or forced expiratory volume in one second (FEV1, the amount of air forcibly exhaled in the first second of a forced breath). APES remains relatively rare and not fully understood, particularly in patients with long-standing stable disease [3,4].

Pulmonary sarcoidosis resolves spontaneously in approximately two-thirds of patients, with radiographic remission observed in many cases within a few years after diagnosis. Nevertheless, 10%-30% of patients follow a chronic or progressive trajectory, with fibrotic changes (scarring of lung tissue) developing in a minority of cases [5]. Although the overall mortality of pulmonary sarcoidosis is low (<10%), disease-related deaths are predominantly attributed to pulmonary fibrosis and cardiac involvement [6].

Notably, most relapses or exacerbations occur within the first 10 years following diagnosis. However, there have been reports of APES occurring more than 20 years later, suggesting the potential for relapse even after long periods of clinical stability [3,7]. Several risk factors have been identified for disease relapse and exacerbation, including bilateral hilar lymphadenopathy (enlargement of lymph nodes on both sides of the hilum, where the bronchi, arteries, and veins enter the lungs), short disease duration, ocular and cardiac involvement, prior corticosteroid use, and pulmonary fibrosis [3,8]. Advanced age at diagnosis and male sex are also associated with worse outcomes [6]. Moreover, patients with sarcoidosis-associated pulmonary fibrosis (SAPF, lung scarring specifically due to sarcoidosis) and traction bronchiectasis (widening of the airways caused by the pulling effect of the surrounding scar tissue) are at an elevated risk of recurrent exacerbations [9].

The underlying biological processes (pathophysiology) of APES are thought to involve persistent immune dysregulation, latent microbial infection (an infection that is present but not causing active symptoms, such as by *Propionibacterium acnes*), and ongoing fibrotic remodeling (i.e., the process of scar tissue formation and reorganization) mediated by specific immune system messengers like T-helper 1 cytokines and transforming growth factor-beta (TGF-β) signaling pathways [10-12]. Despite its clinical significance, APES remains poorly understood, and reports of cases arising decades after initial diagnosis are exceedingly rare.

Herein, we describe a patient who developed an acute pulmonary exacerbation 26 years after the initial diagnosis of stage I sarcoidosis (a classification typically indicating enlarged lymph nodes in the chest without significant lung tissue involvement visible on initial imaging). This case highlights the importance of long-term surveillance, even in patients with initially mild disease and prolonged asymptomatic periods.

## Case presentation

A 59-year-old Japanese man with a history of pulmonary sarcoidosis, initially diagnosed as stage I (characterized by hilar lymphadenopathy without apparent parenchymal involvement on chest radiography at that time) via bronchoscopy 26 years earlier, presented with a four-month history of exertional dyspnea. He had not received any follow-up for respiratory symptoms and was being monitored solely for ocular sarcoidosis. It is important to note that no chest imaging was performed during the 26-year interval between the initial diagnosis and the current presentation, as respiratory follow-up had not been maintained by the patient. Ten days prior to admission, he developed fever and worsening dyspnea at rest, prompting consultation with his previous physician. He had a 39-year smoking history of 20 cigarettes per day, and resided in a 30-year-old wooden apartment building. He did not consume alcohol, was not taking any regular medications, and had no other relevant medical history. Chest auscultation revealed fine crackles in the lower lung fields, and pulse oximetry showed an oxygen saturation of 94% on 4 L/min of nasal oxygen.

**Figure 1:**
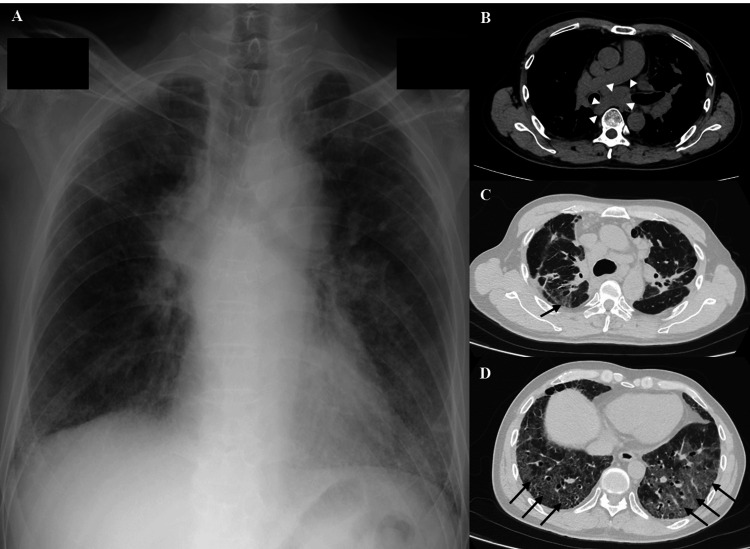
Initial chest radiography and computed tomography (CT) scan (A) Chest radiograph on admission. Diffuse bilateral reticulonodular opacities, predominantly in the lower and mid lung fields, are visible, along with some prominence of the mediastinal silhouette. (B-D) Enlarged mediastinal lymph nodes (white arrow heads), measuring 25 mm in the short axis and 40 mm in the long axis, and diffuse ground-glass opacities (black arrows) are visible in both lung fields. Bilateral lower lobe traction bronchiectasis is also observed, suggesting chronic inflammatory changes.

On admission, an initial chest radiograph showed diffuse bilateral reticulonodular opacities, predominantly in the lower and mid lung fields, along with some prominence of the mediastinal silhouette (Figure 1A). A chest computed tomography (CT) scan revealed enlarged mediastinal lymph nodes and diffuse ground-glass opacities in both lungs, along with traction bronchiectasis in the lower lobes (Figures 1B-1D).

Laboratory studies demonstrated elevated lactate dehydrogenase (LDH), Krebs von den Lungen-6 (KL-6), and soluble interleukin-2 receptor (sIL-2R) levels (Table 1).

**Table 1:** Laboratory findings on admission Plt: platelet; KL-6: Krebs von den Lungen-6; SP-D: surfactant protein D; ACE: angiotensin-converting enzyme; AST: aspartate aminotransferase; ALT: alanine aminotransferase; LDH: lactate dehydrogenase; Alb: albumin; BUN: blood urea nitrogen; Cre: creatinine; IL-2R: interleukin-2 receptor

Test	Value	Reference range
WBC (/μL)	6630	3300-8600
RBC (×10^4^/μL)	472	435-555
Hb (g/dL)	14.9	13.7-16.8
Plt (×10^3^/μL)	45	15.8-34.8
AST (U/L)	31	13-30
ALT (U/L)	27	10-42
LDH (U/L)	346	124-222
Alb (g/dL)	3.6	4.1-5.1
BUN (mg/dL)	11	8-20
Cre (mg/dL)	0.7	0.65-1.07
Na (mEq/L)	141	138-145
K (mEq/L)	3.9	3.6-4.8
Ca (mg/dL)	9.7	8.8-10.1
CRP (mg/dL)	1.17	0.00-0.14
Ferritin (ng/mL)	193	10-250
KL-6 (U/mL)	4622	0-500
SP-D (ng/mL)	920	0-110
ACE (IU/L)	12.3	7.7-29.4
IgG (mg/dL)	1143	861-1747
IgA (mg/dL)	356	93-393
IgM (mg/dL)	22	33-183
IL-2R (U/mL)	1495	121-613
Budgerigar-specific IgG (mg_A_/L)	5	0-8
Dove-specific IgG (mgA/L)	9	0-24
Anti-*Trichosporon asahii *antibody	0.13	0-0.15

Serum autoantibodies were negative. Given the patient’s long smoking history, residential environment (a 30-year-old wooden apartment building), and diffuse ground-glass opacities on imaging, hypersensitivity pneumonitis (HP) was considered in the differential diagnosis. However, a detailed exposure history revealed no clear exposure to common HP antigens such as avian proteins (no feather bedding, down jackets, or direct bird contact reported) or significant molds; no other specific environmental or occupational antigen exposure was identified. Bird-specific IgG antibodies were within normal ranges. These factors, combined with the prominent mediastinal lymphadenopathy and the histopathological confirmation of non-caseating epithelioid granulomas from both lung and lymph node biopsies, strongly favored active sarcoidosis over HP. Cultures and PCR testing for bacteria, fungi, and mycobacteria were also negative.

Bronchoscopy was performed on the day after admission. Bronchoalveolar lavage (BAL) fluid collected from the left B5 bronchus showed a total white blood cell count of 460 × 10³/mL, with lymphocytes accounting for 44% (Table 2).

**Table 2:** Bronchoalveolar lavage (BAL) fluid analysis

Test	Value
WBC (×10^3^/mL)	460
Neutrophil (%)	2
Lymphocyte (%)	44
Histiocyte (%)	50
Eosinophil (%)	4
Basophil (%)	0
CD4 (%)	61.1
CD8 (%)	34.4
CD4/CD8	1.8

**Figure 2:**
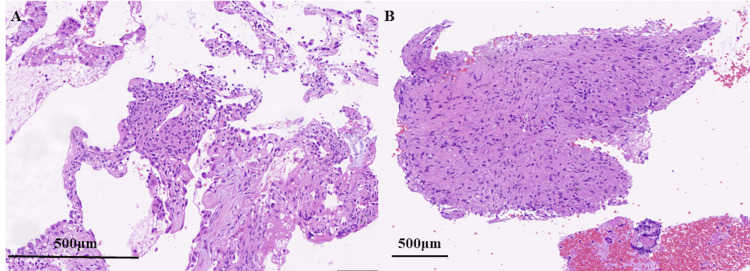
Histopathological findings from bronchoscopy specimens (A) Hematoxylin and eosin (H&E) staining of a transbronchial lung cryobiopsy (TBLC) specimen from the left B8a segment reveals a well-formed non-caseating epithelioid granuloma surrounded by organizing inflammation. (B) Endobronchial ultrasound-guided transbronchial needle aspiration (EBUS-TBNA) from mediastinal lymph node station 7 shows a non-caseating epithelioid granuloma.

Transbronchial lung cryobiopsy (TBLC) from the left B8a segment revealed non-caseating epithelioid granulomas with surrounding organizing inflammation, while endobronchial ultrasound-guided transbronchial needle aspiration (EBUS-TBNA) from mediastinal lymph node station 7 also showed granulomatous inflammation consistent with sarcoidosis (Figure 2).

Cultures and PCR testing for bacteria, fungi, and mycobacteria were negative.

**Figure 3:**
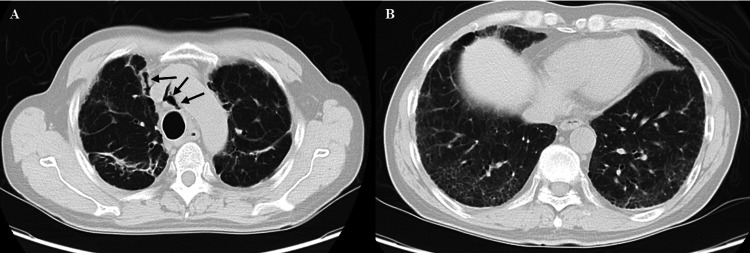
Follow-up chest CT after corticosteroid therapy Significant improvement of ground-glass opacities is observed in both lungs following steroid treatment. However, reticular opacities remain, and new-onset pneumomediastinum is identified (black arrows).

These findings led to the diagnosis of acute pulmonary exacerbation of sarcoidosis. The patient underwent pulse corticosteroid therapy with intravenous methylprednisolone (1,000 mg/day) for three days, followed by oral prednisolone at 70 mg/day (approximately 1 mg/kg/day). No clinical deterioration occurred, and the dose was tapered weekly by 10 mg. By hospital day 24, the patient no longer required supplemental oxygen at rest, although exertional desaturation persisted. Consistent with this clinical improvement, serum inflammatory markers showed a decline by discharge: KL-6 decreased from 4622 U/mL (admission) to 3163 U/mL (discharge), SP-D decreased from 920 ng/mL to 276 ng/mL, and LDH decreased from 346 IU/L to 226 IU/L (Table 1 shows admission values). Pulmonary function tests (PFTs) were not performed during hospitalization due to the patient's initial severe hypoxemia requiring high-flow oxygen therapy and the subsequent development of pneumomediastinum, which posed a risk during forced respiratory maneuvers. A follow-up CT scan showed marked improvement of the ground-glass opacities but also revealed newly developed pneumomediastinum (Figure 3).

A second course of pulse steroid therapy was administered, followed by tapering to 30 mg/day of prednisolone. The patient was discharged on day 45 with home oxygen therapy for use during physical activity. Future treatment strategies were being considered, including the introduction of immunosuppressive agents, given the risk of recurrent exacerbations during steroid tapering.

## Discussion

This case highlights a rare instance of APES occurring 26 years after the initial diagnosis of stage I disease. While sarcoidosis typically follows a self-limiting course, with spontaneous remission observed in up to two-thirds of patients [3,5], this case demonstrates that even after decades of clinical stability, acute exacerbation can occur. The favorable response to corticosteroids in our patient underscores the importance of recognizing and promptly managing late-onset APES to prevent irreversible lung injury. The robust response to corticosteroids observed in this patient is likely attributable to the active granulomatous inflammation, as evidenced by the biopsy findings of non-caseating granulomas and elevated inflammatory markers (sIL-2R, KL-6), which are known to be steroid responsive. While specific prognostic factors for such late-onset APES are not well-defined in the literature due to its rarity, factors associated with relapse or exacerbation in general sarcoidosis include the extent of fibrotic change, degree of initial inflammatory burden, and potentially, the presence of certain comorbidities or triggers. This case highlights that even after prolonged stability, significant steroid-responsive inflammation can occur. The development of pneumomediastinum following initial treatment (Figure 3) is a notable finding. Its etiology in this case is likely multifactorial, possibly attributed to the Macklin effect, where alveolar rupture due to underlying fibrotic lung disease and severe inflammation, perhaps exacerbated by coughing or increased respiratory effort, leads to air dissecting along bronchovascular sheaths into the mediastinum. While a direct complication of steroid therapy is less common, steroid-induced tissue fragility could be a contributing factor in susceptible individuals. The pneumomediastinum was managed conservatively and improved.

Although the majority of pulmonary sarcoidosis exacerbations are reported within 10 years of diagnosis [3,6], a few cases have shown relapse occurring beyond 20 years [7]. In a retrospective Japanese cohort study, the average time to relapse or exacerbation was 8.7 ± 8.3 years, and only a small percentage of patients experienced recurrence after such an extended period [3]. This patient's 26-year asymptomatic period, followed by a sudden onset of respiratory deterioration, makes this a particularly rare clinical course. Additionally, the presence of traction bronchiectasis and fibrotic changes, as observed on CT, may indicate chronic smoldering disease with late reactivation [9]. While the patient was clinically asymptomatic from a respiratory perspective and thus considered 'stable' during the intervening 26 years, the current radiological findings of traction bronchiectasis and fibrotic changes (Figure 1) suggest a slow, subclinical progression of his pulmonary sarcoidosis from the initial stage I classification. The exact timing of this progression cannot be determined due to the lack of interval respiratory-focused follow-up and imaging.

The diagnostic approach in this case was strengthened by the integration of imaging, laboratory, and pathological data. High-resolution CT revealed diffuse ground-glass opacities and mediastinal lymphadenopathy, common in APES [9]. Bronchoalveolar lavage showed lymphocytosis (44%), and TBLC specimens revealed well-formed non-caseating granulomas, consistent with sarcoidosis. Serum KL-6, SP-D, and sIL-2R levels were elevated, further supporting active inflammation. The patient's serum sIL-2R level on admission was 1495 U/mL, markedly elevated and consistent with high disease activity. For context, one study reported a mean sIL-2R level of 1164.3 ± 975.5 U/mL in sarcoidosis exacerbation groups [3], placing our patient's level well within such an active range. Similarly, the KL-6 level of 4622 U/mL was significantly increased, indicative of active pneumonitis, although direct comparative ranges specifically for APES from all literature sources are limited. These findings, along with negative infectious and autoimmune panels, helped exclude other causes such as infection or hypersensitivity pneumonitis, the latter being ruled out by low bird-specific IgG levels [8].

Several risk factors for sarcoidosis relapse and exacerbation have been identified in the literature. These include bilateral hilar lymphadenopathy, short disease duration, ocular or cardiac involvement, and prior corticosteroid use [3,6,8]. Our patient exhibited ocular involvement and had not received systemic treatment in the past, potentially contributing to prolonged disease quiescence. Interestingly, pulmonary fibrosis and traction bronchiectasis are known to predispose patients to APES [9], and recent studies suggest that persistent immune activation, elevated TGF-β levels, and latent infections such as with *Propionibacterium acnes* may contribute to disease reactivation [10-12].

This case reinforces the need for long-term monitoring, even in patients initially diagnosed with mild disease. Patients should be educated about the possibility of late pulmonary complications and advised to seek medical attention if respiratory symptoms develop. At the time of diagnosis, a structured follow-up plan could help identify early signs of relapse. Furthermore, in cases with imaging evidence of fibrotic progression, periodic pulmonary function testing and chest imaging may be warranted to guide early therapeutic intervention. Future research should aim to establish risk stratification models and biomarkers to predict APES and tailor follow-up strategies accordingly.

## Conclusions

This case illustrates that acute pulmonary exacerbation of sarcoidosis can occur even after more than two decades of apparent clinical stability. Although sarcoidosis is often self-limiting, the potential for late-onset respiratory deterioration should not be underestimated. Radiological and pathological findings, in conjunction with laboratory markers, were instrumental in establishing the diagnosis and guiding effective corticosteroid treatment.

Long-term follow-up remains essential for patients with sarcoidosis, particularly those with extrapulmonary involvement or imaging evidence of fibrotic changes. Structured surveillance strategies and patient education are crucial to detect early signs of disease reactivation. This case highlights the need for continued clinical awareness and further research to identify predictive factors and optimize long-term management in sarcoidosis.
